# Epidemiology of Patients Hospitalized for Ocular Trauma in the Chaoshan Region of China, 2001–2010

**DOI:** 10.1371/journal.pone.0048377

**Published:** 2012-10-31

**Authors:** He Cao, Liping Li, Mingzhi Zhang

**Affiliations:** 1 Injury Prevention Research Center, Medical College of Shantou University, Shantou, Guangdong Province, People’s Republic of China; 2 Joint Shantou International Eye Centre, Shantou, Guangdong Province, People’s Republic of China; Medical University Graz, Austria

## Abstract

**Background:**

The burden and pattern of ocular trauma in China are poorly known and not well studied. We aimed at studying the epidemiological characteristics of patients hospitalized for ocular trauma at major ophthalmology departments in the largest industrial base of plastic toys in China.

**Methods:**

A retrospective study of ocular trauma cases admitted to 3 tertiary hospitals in China from 1st January 2001 to 31st December 2010 was performed.

**Results:**

The study included a total of 3,644 injured eyes from 3,559 patients over the 10-year period: 2,008 (55.1%) open-globe injuries, 1,580 (43.4%) closed-globe injuries, 41 (1.1%) chemical injuries, 15 (0.4%) thermal injuries and 678 (18.6%) ocular adnexal injuries. The mean age of the patients was 29.0±16.8 years with a male-to-female ratio of 5.2∶1 (*P* = 0.007). The most frequent types of injury were work-related injuries (1,656, 46.5%) and home-related injuries (715, 20.1%). The majority of injuries in males (56.2%) and females (36.0%) occurred in the 15–44 age group and 0–14 age group, respectively. The final visual acuity correlated with the initial visual acuity (Spearman’s correlation coefficient = 0.659; *P*<0.001). The Ocular Trauma Score also correlated with the final visual acuity (Spearman’s correlation coefficient = 0.655; *P*<0.001).

**Conclusions:**

This analysis provides an epidemiological study of patients who were hospitalized for ocular trauma. Preventive efforts are important for both work-related and home-related eye injuries.

## Introduction

Ocular trauma is a common cause of unilateral blindness [1] and is associated with significant emotional stress as well as numerous emergency room [2] and outpatient visits [3]. Worldwide, 55 million eye injuries restricting activities more than one day occur each year; there are approximately 1.6 million blind people from injuries, an additional 2.3 million people with bilateral low vision from this cause [4]. Each year, more than 2.5 million eye injuries occur in the United States, and 50,000 people permanently lose part or all of their vision [5], [6]. The incidence of eye injuries may be higher in developing countries [7], [8]. In addition, the rates at which eye injuries require hospitalization have ranged from 4.9 to 89 per 100,000 [5], [9]–[16], [25]. Despite the heterogeneity of the results, these studies provide important information regarding the burden of eye injuries.

Clinical and epidemiological ocular trauma studies have been described in the United States [5], [9], [12], [13] and other developed countries [10], [14], [15], [17]–[25]. Information on its epidemiology from developing countries is also available [7], [26]–[30], but the burden and pattern of injuries in developing countries are poorly known and not well studied [31]. China still lacks complete eye injury statistics and authoritative epidemiological data.

The Chaoshan region is located in the eastern part of Guangdong Province, which is the largest industrial base for plastic toy production in China. This study included all hospitalized patients with ocular and orbital trauma in the Ophthalmology Departments of three major tertiary hospitals from 1st January 2001 to 31st December 2010. This study may help to identify contributing factors associated with highly industrialized environments and to find preventive methods for minimizing such disabling injuries. During the study period, the population was stable, and there were no significant changes in gender or age structure. Hence, these data may be representative of any of the surrounding coastal industrial regions.

## Methods

The research described herein adhered to the tenets of the Declaration of Helsinki. All medical records were anonymous and no patient information could be extracted except for research purposes. The informed written consent was given by the patients and the next of kin, caregivers or guardians on behalf of the minors/children participants involved in our study. The procedure was approved by the Ethics Committee of the Medical College, Shantou University, the Joint Shantou International Eye Center, the First Affiliated Hospital of Shantou University and Shantou Center Hospital. The region of investigation included three cities and covered approximately 10346 square km. According to the National Statistical Yearbook 2010, the annual average population was 12,989,800 (95% confidence interval: 12,328,300–13,471,900) during the study period. The regional economic sectors include small-scale industries, agriculture, fishing, trade and tourism. The patients were classified into six age groups (0–14, 15–29, 30–44, 45–59, 60- years) based on the criteria used in the China Injury Surveillance System and the National Statistical Yearbook. The Ophthalmology Departments of the three tertiary hospitals offer excellent eye care services and both emergency and specialized care to hospitalized patients of all ages with specific and complicated ocular or orbital diseases and houses a 24-hour Ophthalmic Emergency Department. This setup provided the opportunity to analyze ocular trauma in a well-defined study region. Ocular trauma was defined as any injury affecting the eye or adnexa that required hospital admission and had a principal or secondary discharge diagnosis from the International Classification of Diseases, Tenth Revision, Clinical Modification (ICD-10-CM). Completed records from 3559 patients were classified by the standardized international classification of ocular trauma (Birmingham Eye Trauma Terminology, BETT) [32], [33]. The extracted patient data included age, gender, occupational categories, the place of residence, the date and cause of injury, the initial and final best-corrected (Snellen) visual acuity, the clinical diagnosis, the primary and secondary treatments, the duration of hospitalization and follow–up. We also utilized the Ocular Trauma Score (OTS) [32], [34] to evaluate the final visual outcome. We classified the data into six groups based on the place where eye injuries occurred: work-related injuries, home-related injuries (falls or penetrating objects, toy bullets, struck against, etc.), school-related injuries (stationary learning, such as injuries due to pencils, knives, etc.), sport-related injuries, road traffic-related injuries, violence-related injuries, and other various outdoor activity-related injuries that cannot be classified into the former categories (such as injuries due to firecrackers, animal assaults, etc.). The initial visual reading was the best corrected Snellen visual acuity (BCVA) in the affected eye at the time of presentation. The final visual acuity was taken during the last follow-up visit (uniform duration of follow up: 1 year). Excluded from the analysis were 26 uncompleted case records and 42 cases that were admitted for treatment but were from outside regions.

### Statistical Analysis

The data were analyzed using SPSS version 17.0 (SPSS, Inc., Chicago, IL, US). Eye injury incidence rates were calculated for each year (2001–2010). Frequency distributions were created for injury types and causes. Statistical analyses of the quantitative data, including descriptive statistics and parametric and non-parametric comparisons, were performed for all variables. Frequency analyses were performed using Pearson’s Chi-squared test. A one-way analysis of variance (ANOVA) was used to evaluate differences in parametric variables. Categorical evaluations were performed for the numeric scores representing the likelihood of the final visual acuity in the OTS study.

Correlation analyses (between the initial and final visual acuity and OTS and the final visual acuity) were performed using Spearman’s test. All *P*-values reported are two sided, and a value less than 0.05 was considered statistically significant.

## Results

### Characteristics of Demographics and Diagnosis

This study included data from 3644 injured eyes from 3559 patients over a 10-year period. The age of the patients ranged from 2 months to 88 years with a mean and standard deviation of 29.0±16.8 years. Within the patient population, 2982 (83.8%) were males, and 577 (16.2%) were females (*P* = 0.007; Pearson’s Chi-squared test), yielding a male-to-female ratio of 5.2∶1. The mean ages were 31.0±16.2 years for the males and 18.5±15.7 years for the females (*P = *0.005; ANOVA test). Most male patients (50.4%) in this study were blue collar workers (physical laborers) under 44 years old, whereas the females who presented ocular trauma (36.0%) were more likely to be children aged 14 years or younger. There was no significant difference in the frequency of right vs. left eye injuries (1904 right vs. 1740 left, 85 bilateral eye injuries). The incidence of open-globe injuries (2008 eyes, 55.1%) was higher than closed-globe injuries (1580 eyes, 43.4%). Six hundred seventy-eight patients (18.6%) had ocular adnexal injuries. Based on national population census data, the average annual hospitalization rate due to eye injuries for our health district was 27.7 per 100,000 (95% CI, 26.4–28.9) (Table 1).

**Table 1:** Characteristics of patients hospitalized with eye injury diagnoses over a 10-year period (1stJanuary 2001–31stDecember 2010).

Total eye injuries	3644
Total patients	3559
Annual hospitalized injuries incidence (per 100,000)	27.7
Right/left eye	1904/1740
Open/closed globe	2008/1580
Male/female	2982/577
Age (years, Mean ± SD^a^)	
Total	29.0±16.8
Male	31.0±16.2
Female	18.5±15.7
Mean duration of hospitalization (days)	8.4±10.3
Mean duration of follow up (months)	12.6±1.5
Diagnosis	ICD-10-AM Code
Open-globe injuries	2008
Penetrating	1823
IOFB^b^	509
Perforating	12
Rupture	272
Closed-globe injuries	1580
Contusion of the eye and adnexa	1573
Lamellar laceration	7
Chemical burn confined to the eye and adnexa	41
Thermal burn confined to the eye and adnexa	15
Adnexa injuries of globe	678
Orbital wall fractures	76
Lacrimal apparatus injury	371
Eyelid injury	194
Injury to the ocular motor nerves	27
Conjunctiva injury	10

ICD-10-AM Code = International Classification of Diseases, Tenth Revision.

SD ^a^: Standard Deviation.

IOFB ^b^ = Intraocular Foreign Body.

### Injury Types Distributions by Age and Gender

**Figure 1:**
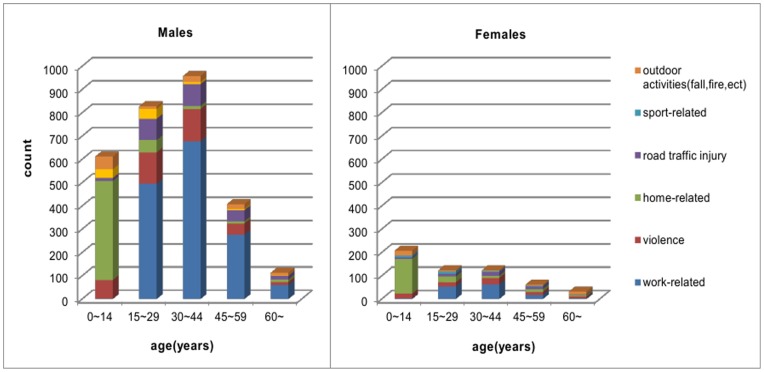
Frequency of types of eye injuries by age and gender. The majority of injuries (56.2%) occurring in males were distributed in the 15–44 age groups; the most frequent injury cause were related to work (70.0%), violence (16.3%), and road traffic (6.2%). The majority of injuries (36.0%) occurring in females were distributed in the 0–14 age group; the most frequent injury cause were related to the home (72.1%), violence (9.6%), and sports (3.8%).

The most frequent injury types among all cases were work-related injuries (46.5%), home-related injuries (20.1%), violence-related injuries (14.0%) and road traffic injuries (8.8%). There was a correlation between injury cause and gender (*P* = 0.001; Pearson’s Chi-squared test). The majority of injuries (56.2%) occurring in males were distributed in the 15–44 age group, for which the most frequent injury types were work-related injuries (70.0%), violence (16.3%), and road traffic injuries (6.2%). The majority of injuries (36.0%) occurring in females were distributed in the 0–14 age group, for which the most frequent injury types were home-related injuries (72.1%), violence (9.6%), and sports-related injuries (3.8%). Violence accounted for 14.0% of all injuries, 13.9% in males and 14.7% in females. The sources of injury were broad and varied (Figure 1).

### Causative Agents Resulting in Eye Injuries

There was a wide variety of causative agents that resulted in eye injuries (Table 2). The agent of injury was primarily work related (46.5%), including metal fragments/nails (883, 53.3%), wire/steel (333, 20.1%) and stone (252, 15.2%). For home-related injuries, knives/scissors were the leading agents of eye injuries (506, 70.8%), while fingers/fists were the main agents (191, 38.2%) for cases of violence-related injuries. Eye protection was present for only two patients (one was a metal welder, and the other was a victim of violence) (Table 2).

**Table 2:** Causative agents of the most frequent injury categories by gender.

Inciting agent	Male (%)	Female (%)	Total (%)
Work related	1513 (50.7)	143 (24.8)	1656 (46.5)
Metal fragments/nails	829	54	883
Wire/steel	302	31	333
Stone	226	26	252
Grinding wheel	91	19	110
Chemical burn	40	1	41
Thermal burn	13	2	15
Woody debris	12	10	22
Home related	512 (17.2)	203 (35.2)	715 (20.1)
Knives/scissors	355	151	506
Toys bullets	114	21	135
Hammer	27	19	46
Fall	16	12	28
Violence	415 (13.9)	85 (14.7)	500 (14.0)
Finger/fist	166	25	191
Gun shot	78	11	89
Sticks/twigs	68	49	117
Glass bottle	66	0	66
Knives/scissors	37	0	37
Road traffic injuries	262 (8.8)	50 (8.7)	312 (8.8)
Motorcycle	226	36	262
Vehicle	36	14	50
Total	2702 (90.6)	481 (83.4)	3183 (89.4)

### Presentation Interval and Injury Time

**Figure 2:**
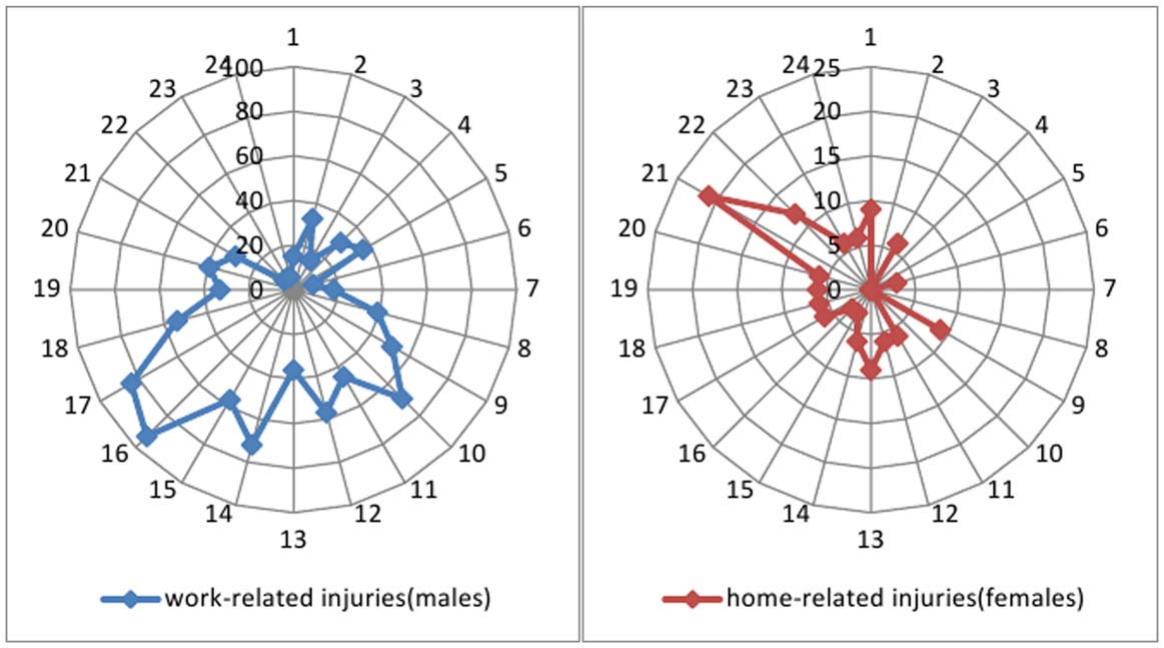
Incidence times of work-related and home-related eye injuries in males and females, respectively, plotted over a one-day period. Work-related eye injuries in males were spread throughout the day and night, with most injuries occurring during 16∶00–17∶00 (12.3%); home-related injuries in females primarily occurred during 21∶00–22∶00 (16.4%). The radial numbers are a count of patients for the entire 10-year period and the circumferential numbers are the hours of the day.

We also evaluated the time interval from the point of injury to arrival at the clinic: 2293 patients (64.4%) arrived in the emergency room in less than 6 hours, 732 (20.6%) within 6 to 12 hours after injury, 195 (5.5%) within 12 to 24 hours after injury, and 339 (9.5%) arrived more than 1 day after the injury. Therefore, 9.5% of patients took more than 24 hours to seek medical care after their injury (3.6% were workers, and 5.9% were children). There was a significant difference in the final visual acuity between the patients who arrived in the emergency room within 24 hours and those who arrived more than 24 hours after the injury (*P*<0.01; Pearson’s Chi-squared test). A presentation interval with 24 hours or longer after injury means a worse prognosis. The eye injuries occurred throughout the day and night, with most work-related injuries in males occurring from 16∶00 to 17∶00 (188 cases, 12.3%) and most home-related injuries in females from 21∶00 to 22∶00 (40 cases, 16.4%). There was no significant variation in the day of the year or the season in which the injuries occurred (Figure 2).

### Management

A total of 809 (22.2%) patients were managed conservatively on medications, and the remaining 2835 (77.8%) required additional procedures. Ocular wall repair (1349, 37.0%) and lens extraction (567, 15.6%) were the most commonly required additional procedures. Eighty-five (2.3%) eyes exhibited anterior chamber washout following severe hyphema and anterior chamber foreign bodies. Posterior vitrectomies were required for 266 (7.3%) eyes with intravitreal foreign bodies (86 eyes), vitreous hemorrhages (64 eyes), retinal detachments (60 eyes) and endophthalmitis (56 eyes). Despite optimal follow-up, the functional results after combined anterior and posterior segment injuries were discouraging: only 19.2% of the eyes attained a BCVA of 0.3 or better after primary wound closure and secondary surgery for reconstruction. However, 31.8% of eyes with injuries limited to the anterior segment achieved a BCVA of 0.3 or better (*P*<0.01, Pearson’s Chi-squared test). In 52 (1.4%) no light perception (NLP) eyes, enucleation was carried out for globe rupturing and uncontrolled endophthalmitis. In addition, 371 (10.3%) eyes with a canalicular fracture received anastomosis, and 56 (1.5%) orbital fracture repairs were performed due to significant enophthalmos and persistent diplopia (Table 3).

**Table 3:** Non-surgical and surgical management reports from initial presentation to final follow up in eye injury cases.

Management	Frequency	Percentage (%)
Non-surgical	809	22.2
Surgical	2835	77.8
Ocular wall repair	1349	37.0
Lens extraction	567	15.6
Posterior vitrectomy	266	7.3
Anterior chamber washout	85	2.3
Scleral Buckle	49	1.3
Keratoplasty	0	0
Enucleation	52	1.4
Glaucoma surgery	40	1.1
Canalicular anastomosis	371	10.3
Orbital fracture repair	56	1.5
Total	3644	100

### Correlation between Visual Outcome and OTS

There was a significant difference in final visual acuity between open- and closed-globe injuries (*P*<0.001; Pearson’s Chi-squared test). The closed-globe injuries appeared to have a better prognosis because 1282 (71.2%) open-globe injured eyes had the final visual acuity lower than 0.3, while 947 (67.6%) closed-globe injured eyes had the final visual acuity equal to or better than 0.3. A comparison of the final visual acuity and the presenting visual acuity is shown in Table 4. The final visual acuity was 20/40 or better in 1092 eyes (30.0%), 20/100–20/50 in 550 eyes (15.1%), and 20/100 or less in 1354 eyes (37.2%). Two hundred fifty-six eyes (7%) had a final visual acuity of no light perception, and 3219 eyes with an initial visual acuity number were classified in OTS categories 1 through 5. Those patients with no light perception for their initial acuity had a poor prognosis. The initial visual acuity correlated with the final visual acuity (Spearman’s correlation coefficient = 0.658; *P*<0.001). The OTS also correlated with the final visual acuity (Spearman’s correlation coefficient = 0.655; *P*<0.001) (Table 5).

**Table 4:** A comparison of final visual acuity and presenting visual acuity.

Visual acuity	At presentation		At last follow up	
	Frequency	Percentage (%)	Frequency	Percentage (%)
NLP	236	6.5	256	7.0
4/200-LP	1645	45.1	899	24.7
19/100–5/200	421	11.6	455	12.5
20/50–20/100	371	10.2	550	15.1
≥20/40	546	15.0	1092	30.0
Others	425^a^	11.6	392^b^	10.7
Total	3644	100	3644	100

(Spearman’s correlation coefficient = 0.659, *P*<0.001).

425^a^: includes the patients who were too young to receive the visual acuity examination and those with presenting symptoms that were too serious to apply to the visual acuity examination.

392^b^: includes the patients who were too young to receive the visual acuity examination, those transferred to another health care facility, receiving home health care, not adhering to medical advice or with missing/unrecorded data, and death.

**Table 5:** Correlation of the final visual acuity category with the Ocular Trauma Score in 3219 eyes.

OTS		Final visual acuity category					
Raw score	Category	NLP	LP-4/200	5/200–19/100	20/100–20/50	≥20/40	Total
**0–44**	**1**	**135**	**148**	**63**	**12**	**9**	**367**
**45–65**	**2**	**115**	**429**	**125**	**113**	**90**	**872**
**66–80**	**3**	**6**	**291**	**217**	**311**	**394**	**1219**
**81–91**	**4**	**0**	**13**	**26**	**93**	**239**	**371**
**92–100**	**5**	**0**	**18**	**24**	**21**	**327**	**390**
**Total**	**256**		**899**	**455**	**550**	**1059**	**3219**

(Spearman’s correlation coefficient = 0.655, *P*<0.001).

## Discussion

Estimates of the rate of eye injury are highly dependent on the definition and the source of data [35]. Hospital discharge data provide a useful source of such information. To our knowledge, this is the most current study that has examined the epidemiology of hospitalized eye injuries over a 10-year period in China. Our findings indicate that ocular trauma is a significant cause of visual loss in this population. Preventive efforts are important for both work-related and home-related eye injuries.

### Characteristics of Demographics and Diagnoses

This study estimates that the annual incidence rate of hospitalized eye injury is 27.7 per 100,000. This rate is higher than that reported by Pamela L Owens et al. in the United States in 2011 (6.5 per 100,000) [35], Ayman Saeed et al. in Ireland in 2010 (18.0 per 100,000) [14], Chua D et al. in Singapore in 2011 (12.6 per 100,000) [36], Salvatore Cillino in Italy in 2008 (4.9 per 100,000) [25], and less than Raymond S et al. in Australia in 2010 (53.3 per 100,000) [10]. A bias of underestimating the true incidence of ocular trauma may have occurred due to the loss of many of the minor trauma cases who may have sought care for eye injuries in other hospitals out of this region and of cases of polytraumatized patients. The risk to the large local population base cannot be accurately determined, which is a limitation of this study. This result reflected the higher incidence rate of hospitalized eye injuries and disease burden in our study region. The incidence of open-globe injuries was higher than closed-globe injuries. This finding differs from the result reported by Ojabo CO in Nigeria [37] and Pandita A in New Zealand [38], who reported that closed-globe injuries were more common than open-globe injuries. These discrepancies could likely be attributed to the higher proportion of occupational injuries from sharp, or penetrating injuries. The patients in this study did not use proper eye protection when conducting hazardous tasks. Further research to better understand the poor compliance with protective eyewear regulations is recommended.

### Injury Types Distributions by Age and Gender

Age and gender were found to correlate with the susceptibility to ocular trauma. However, the mean age for ocular injury in this study was 29.0 years, which corresponds to most other studies in which a mean age of approximately 30 years has been reported [39]–[45]. This is likely due to the work-related injuries that contributed to the largest portion of injuries (46.5%). The percentage of children aged 14 years or less was 21.1 (95% CI 18.9–23.4) during the study period based on the National Statistical Yearbook 2010. Due to the prosperity of the local plastic toy industry, there are many household toy manufacturing plants. In this study, children often assisted in toy production, and they might have worked and played with toys of substandard quality, resulting in a significantly larger pediatric portion in our study population (23.6%) compared to other studies [41], [44]. These two types of patients are mostly exposed to sharp instruments: workers with machines and rough instruments and children with various sharp instruments and toys at home or in school. Similar to other studies [41]–[43], males constituted 83.8% of the patients, with a male-to-female ratio of 5.1 to 1.

### Causative Agents Resulting in Eye Injuries

Metal fragments/nails remain the leading (883, 53.3%) agents that cause eye injury among all work-related injuries according to previous reports in the literature [44]–[48]. The local work tasks include grinding, welding, hammering, drilling, carpentry, metal cutting and nailing. These activities commonly involve high-powered tools that generate metal fragments/nails at high velocities and often have devastating effects on the eye. It is recognized that males have a relatively higher tendency for work-related, violence-related and road traffic-related eye injuries. Among transportation-related injuries, motorcycle accidents are one of the highest risks for eye injuries. A large-scale prospective study of violence-related eye injuries is needed.

### Presentation Interval and Injury Time

The current study has shown that work-related eye injuries occur during multiple daily peaks in males, with most injuries occurring near the end of the day (16∶00–17∶00); night operation was also a risk factor. This finding differs from the study performed by Justin M et al. in the United States in 2010 [47], which showed a double peak during the course of a day. These injury times correlate with regular daily activity. Other contributing factors include worker fatigue and overtime, which were also reported to increase the risk of occupational eye injuries by Blackburn J et al. [48]. Workers could benefit from better training to recognize fatigue and strategies to prevent fatigue from impacting their work, especially while completing hazardous tasks in the afternoon. Another explanation may be the workflow at job sites; more hazardous work may be undertaken at these times. More research is needed to better understand the significance of these time-related circumstances with respect to eye injuries. Additionally, home-related eye injuries in females most often occurred during 21∶00∼22∶00. Female children aged 14 years or younger represented 73.9% (150/203) of the injuries, reinforcing the need for prevention of childhood injuries within the home and at night. Delayed presentation is also a matter of concern. After injury, 9.5% of patients took longer than 24 hours to seek medical care. Poverty, the absence of decision-makers at home and a lack of awareness among parents can hamper timely management of ocular injuries in our study region. Further studies investigating these barriers are recommended as most of the patients in this study came from poor socioeconomic groups.

### Management

The principal surgical management involved in ocular injuries among our sample suggests that more damage is inflicted to the anterior segment than the posterior segment of the eye (1349 ocular wall repairs, 567 lens extraction, and 85 anterior chamber washouts). The training modules for mid-level eye care personnel and general ophthalmologists in the study region should emphasize the anterior segment of the eye, types of eye injuries, appropriate precautions and first-aid management of the eye. Among the 678 adnexa injuries, 215 were complicated open-globe injuries, 445 were closed-globe injuries and 18 were chemical/thermal burns to the eyeball. The high rate of orbital fracture repair (56/76, 73.7%) in our study demonstrated that ocular trauma was a frequent cause of skull fractures requiring a neurosurgical approach.

### Correlation between Visual Outcome and OTS

In our study, open-globe injuries exhibited poorer visual prognoses than closed-globe injuries. The most common open-globe injuries were anterior penetrating injuries and were associated with good visual prognosis, whereas open injuries that involved the posterior globe often accompanied retinal detachment, severe vitreous hemorrhage or endophthalmitis which are associated with poor prognoses. These findings are consistent with other studies [47], [49]–[51]. We analyzed the OTS distribution in the two types of globe injuries. The results showed that 1000 (49.8%) eyes had open-globe injuries distributed in the lower OTS level (1 to 2). 1177 eyes (74.5%) had closed-globe injuries distributed in the higher OTS level (3 to 5). Higher OTS scores tend to indicate a better prognosis. Good initial vision and a high OTS were statistically correlated with good final vision and remain the most important prognostic factors when counseling patients after injury.

This study had several limitations. First, we were unable to follow the long-term-associated morbidities and outcomes using patient medical records. Second, because a nationwide eye injury surveillance system has not been established, the total number of hospitalized eye injuries may be an underestimate. This study excluded patients who might have suffered minor self-resolving ocular trauma or those who were treated in community primary care facilities, physicians’ offices, and the ophthalmology wards of secondary hospitals. Because the above-mentioned medical facilities did not have ophthalmic microsurgical equipment and could not deal with complex medical treatment or perform surgical procedures for ocular trauma, the patients requiring primary care in these hospitals would be referred to one of the three tertiary hospitals in our study. The risk to the local population cannot be accurately determined, which is also a limitation of this study. In addition, there might be some individuals with ocular trauma who had not sought any medical treatment for unknown reasons. We should also consider the loss of many of the minor trauma cases who might have sought care for eye injuries in other hospitals out of this region and of cases of polytraumatized patients. Lastly, there is a geographical bias that is inherent to all ophthalmic trauma study designs. However, these limitations do not significantly affect the major findings of this study. The comprehensive nature and detailed data from each patient’s history, clinical presentation and examination findings ensure that the spectrum and distribution of hospitalized ocular traumas in the coastal urban setting are well represented.

In summary, eye injury research and prevention can be further aided by a national collaborative registry of eye injuries in China. A long-term nationwide database of all ophthalmic injuries is recommended.
